# Sulfur Administration in Fe–S Cluster Homeostasis

**DOI:** 10.3390/antiox10111738

**Published:** 2021-10-29

**Authors:** Leszek Rydz, Maria Wróbel, Halina Jurkowska

**Affiliations:** Medical Biochemistry, Faculty of Medicine, Jagiellonian University Medical College, 7 Kopernika St., 31-034 Kraków, Poland; leszek.rydz@uj.edu.pl (L.R.); mtk.wrobel@uj.edu.pl (M.W.)

**Keywords:** Fe–S cluster, iron–sulfur protein, rhodanese, 3-mercaptopyruvate sulfurtransferase, oxidative stress

## Abstract

Mitochondria are the key organelles of Fe–S cluster synthesis. They contain the enzyme cysteine desulfurase, a scaffold protein, iron and electron donors, and specific chaperons all required for the formation of Fe–S clusters. The newly formed cluster can be utilized by mitochondrial Fe–S protein synthesis or undergo further transformation. Mitochondrial Fe–S cluster biogenesis components are required in the cytosolic iron–sulfur cluster assembly machinery for cytosolic and nuclear cluster supplies. Clusters that are the key components of Fe–S proteins are vulnerable and prone to degradation whenever exposed to oxidative stress. However, once degraded, the Fe–S cluster can be resynthesized or repaired. It has been proposed that sulfurtransferases, rhodanese, and 3-mercaptopyruvate sulfurtransferase, responsible for sulfur transfer from donor to nucleophilic acceptor, are involved in the Fe–S cluster formation, maturation, or reconstitution. In the present paper, we attempt to sum up our knowledge on the involvement of sulfurtransferases not only in sulfur administration but also in the Fe–S cluster formation in mammals and yeasts, and on reconstitution-damaged cluster or restoration of enzyme’s attenuated activity.

## 1. Introduction

**Figure 1:**
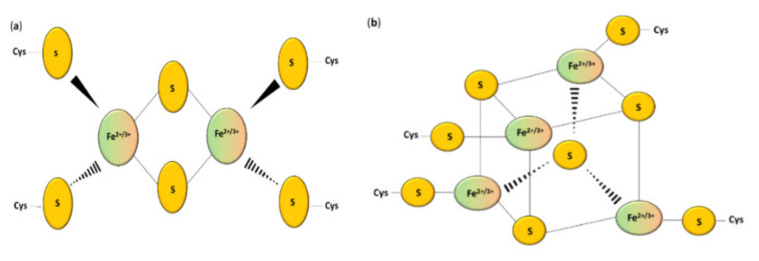
Structure of the most common Fe–S clusters: (**a**) [2Fe–2S]; (**b**) [4Fe–4S] clusters [10].

Sulfur is an important biological element. Various oxidation states of sulfur can occur, ranging from S^2−^ (sulfide) to S^6+^ (sulfate) [1]. Sulfur can be incorporated into proteins, carbohydrates, and lipids participating in many cellular processes, including signaling and redox homeostasis [2]. The most important source of this chemical element are the sulfur-containing amino acids L-cysteine and L-methionine [3]. The sulfur pool can be divided into the stable form (for example L-cysteine and L-methionine) and the labile form. The labile form of sulfur can be further divided into sulfane sulfur (S^0^) and acid-labile sulfur [4]. Hydrogen sulfide (H_2_S) can be released from both labile pools of sulfur under specific conditions [5]. Sulfur, which is a part of metal–sulfur clusters, belongs to the acid-labile group [6]. However, sulfur is not likely to be present inside the cells in the “free” S^2−^ form [7]. Non-heme iron ions can be connected with inorganic sulfur in the polymetallic clusters of proteins, which are called the iron–sulfur (Fe–S) proteins [8]. The iron ions are coordinated via the thiol groups of cysteinyl residues of this peptide [9] and bridged by inorganic sulfide [10] (Figure 1). It is possible that one or more coordinating ligands are changed from the original one to (1) other amino acids, (2) a non-sulfur-based ligand, or (3) another thiolate donating ligand [11].

## 2. Iron–Sulfur Clusters and Iron–Sulfur Proteins

### 2.1. Fe–S Clusters

Iron–sulfur clusters (Fe–S clusters) were firstly discovered around the 1950s [12]. Nowadays, Fe–S clusters can be spotted in every kingdom of life [13]. Moreover, elements of the cluster biogenesis machinery were detected not only in human mitochondria but also in plant mitochondria and even in mitosomes or hydrogenosomes of anaerobic organisms [14]. The primary task of iron ions results from their ability to change their oxidation state from Fe^2+^ to Fe^3+^, contrary to sulfur that is also a part of clusters and is always present in S^2−^ oxidation state [15,16].

Iron–sulfur clusters can be divided into various geometric and stoichiometric forms in which the number of iron and sulfur atoms involved in clusters changes [17]. The simplest [Fe–S] cluster consists of one iron ion bound to a polypeptide by L-cysteine residues [10]. Rhombic [2Fe–2S] (Figure 1a) and cubane [4Fe–4S] clusters (Figure 1b) are the most prevalent groups [7]. Square-like cubane-type iron–sulfur clusters [4Fe–4S] can be produced from two units of [2Fe–2S] clusters [16], and this reaction can be reversed under specific conditions [18]. The loss of one of the atoms results in a non-symmetrical [3Fe–4S] or [8Fe–7S] structure [19,20]. Thus, the iron–sulfur clusters can undergo various transformation processes such as conversion, coordinating ligand exchange, and degradation in oxidative stress [21,22].

### 2.2. Fe–S Proteins

**Figure 2:**
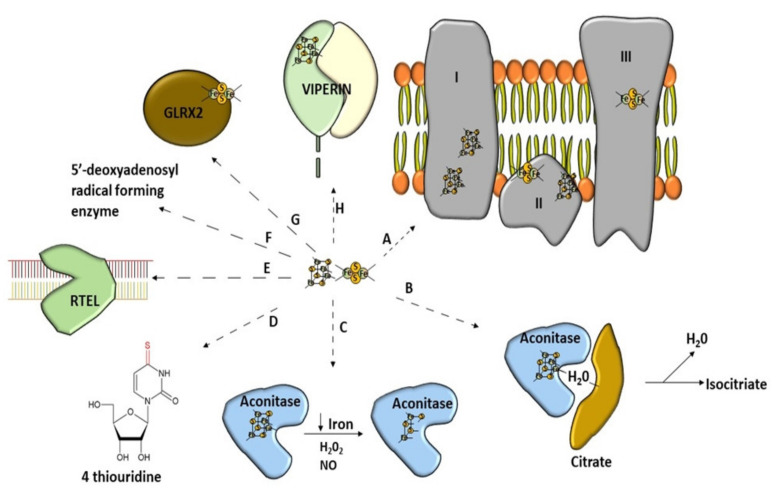
Wide range of use of [Fe–S] clusters. (A) Mitochondrial respiratory chain–NADH dehydrogenase (complex I), succinate dehydrogenase (complex II), Cytochrome bc1 (complex III). (B) Non-redox catalysis (aconitase), (C) regulatory function of aconitase after oxidative stress, (D) involvement in tRNA modification, threonylcarbamoyladenosine tRNA methylthiotransferase (CDKAL1), (E) participation of Fe–S cluster in genome integrity; RTEL (helicase-nuclease DNA2 and regulator of telomere length 1), (F) molybdenum cofactor biosynthesis protein 1 (MOCS1A, a member of the S-adenosylmethionine (SAM)-dependent enzyme family) is an enzyme using the Fe–S cluster to generate a 5′-deoxyadenosyl radical, (G) glutaredoxins with Fe–S clusters are involved in Fe–S cluster synthesis, (H) antiviral proteins using Fe–S cluster to perform their activity (Viperin).

There is a growing list of enzymes that function as Fe–S proteins in various organisms from bacteria to eukaryotes [23,24] (Figure 2). Such proteins can be equipped with different kinds of clusters [25] or include more than one type [26]. All Fe–S proteins perform a wide range of tasks [27]. Human proteins, which include the Fe–S clusters in their structure, can be found mostly in mitochondria but also in the cytosol and even nucleus [28]. Firstly, they are playing the role of a carrier of electrons in the mitochondrial respiratory chain [9,29,30]. Furthermore, the Fe–S clusters are often components of the active site of proteins participating in non-redox catalysis [31]. The Fe–S proteins can also play a role as regulatory agents and protein stabilization factors [8]. They are highly vulnerable to oxidative stress and iron shortage [15]. Therefore, some regulatory proteins recruit the Fe–S clusters in order to better sense O_2_ or to respond to superoxide stress [15]. Furthermore, cells use proteins equipped with the Fe–S clusters to positively affect DNA transcription to regulate iron homeostasis [32,33]. Other peptides can also negatively affect DNA transcription when Fe–S cluster synthesis is sufficient [34]. The Fe–S clusters are cofactors of proteins involved in maintaining genome integrity [35]. DNA-binding proteins have the Fe–S clusters in their structure, and thus their activity is granted [19,36] (Figure 2). They are involved in tRNA modification [7,37]. The Fe–S clusters are also involved in the generation of 5-deoxyadenosyl radicals from S-adenosyl-L-methionine (SAM) [38]. Interestingly, this feature is useful in immunity [39]. Many glutaredoxins, which take part in the Fe–S cluster formation, contain the Fe–S clusters [40]. Most of them can coordinate at least one cluster [41]. The recently discovered activities in which iron–sulfur clusters may be participating are disulfide reduction and sulfur donation [16].

## 3. Iron–Sulfur Cluster Synthesis

L-cysteine is the source of sulfur for the Fe–S cluster synthesis in the overwhelming majority of cases, other sulfur species, such as sulfide are rarely involved [42]. The Fe–S cluster synthesis route is highly conserved among species and consists of a high number of specific proteins [43]. The cluster assembly can take place both in mitochondria and cytosol in Eucaryotes [44]. Fe–S cluster assembly pathways consist of two general events: (1) cluster assembly on the scaffold protein; (2) distribution and final insertion into apo-protein [45]. As far as mitochondrial synthesis and assembly of the Fe–S cluster are concerned, there are three separate systems named ISC (iron–sulfur cluster), SUF (sulfur utilization) [46], and NIF (nitrogen fixation) [47]. The most known system is ISC [10]; this route is the most important biogenesis pathway of the mitochondrial Fe–S clusters under normal conditions [48]. The SUF system is the most ancient of all the Fe–S assembly systems [49]. It is very similar to ISC in many ways [32]. It plays an essential role in viability in SUF-dependent organisms [49]. The components of this pathway (*E. coli* scaffold protein) seem to be more stable under severe conditions [32]. Moreover, the expression of components of this pathway is increased during iron depletion and oxidative stress [47]. Therefore, under adverse conditions the SUF machinery can cover all the Fe–S cluster demands [50]. The SUF system seems to be changing over time since it originated [49]. On the other hand, the NIF system is a dedicated machinery for producing the Fe–S clusters of the nitrogenase protein in *Azotobacter* nitrogen-fixing bacteria [47].

### 3.1. Mitochondrial Iron–Sulfur Cluster Synthesis

The ISC Fe–S cluster synthesis in mitochondria can be precisely divided into four steps detailed later in this manuscript [51]. Starting from the first, there is the [2Fe–2S] cluster de novo synthesis [35]. A main component of assembly system in mitochondria is a specific sulfur delivery enzyme named cysteine desulfurase (NFS1) [52,53]. A further, scaffold protein (ISCU2) [54,55]; a hypothetical iron donor called frataxin (FXN) [56]; an electron donor named ferredoxin (FDX1) [57]; glutaredoxin 5 monothiol (GLRX5)-transfer protein, a specific subset of the thioredoxin (Trx) superfamily [51,58]; and a specific chaperone/co-chaperone complex are required [59].

#### 3.1.1. Early Step of Mitochondrial Fe–S Cluster Synthesis

**Figure 3:**
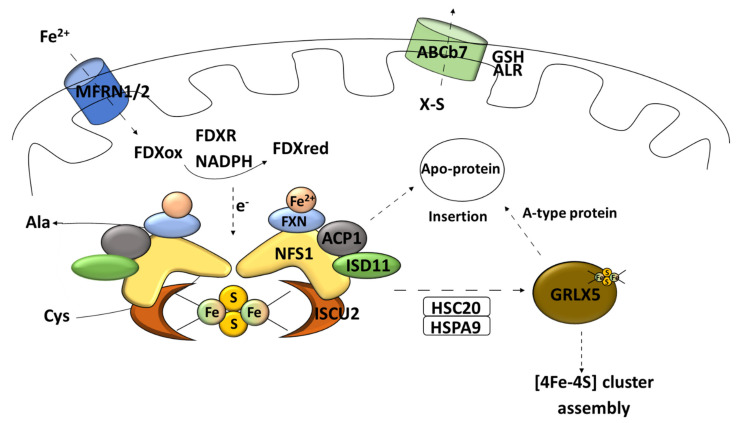
Early stage of mitochondrial Fe–S synthesis. NFS1, cysteine desulfurase; ISD11, LYR protein; ACP1, acyl carrier protein; ISCU2, scaffold protein; MFRN1/2, carrier protein mitoferrin 1 and 2; FXN, frataxin; FDXR, mitochondrial ferredoxin reductase; Cys, L-cysteine; Ala, L-alanine; HSPA9, mortalin/mitochondrial 70 kDa heat shock protein; HSC20, iron–sulfur cluster co-chaperone protein HscB; GLRX5, monothiol glutaredoxin 5; GSH, reduced glutathione; ALR, mitochondrial FAD-linked sulfhydryl oxidase; ABCb7, ATP-binding cassette (ABC) transporter family.

Cysteine desulfurase [EC 2.8.1.7] in the form of homodimers uses L-cysteine as the main source of sulfur for iron–sulfur cluster synthesis [60]. It is a pyridoxal phosphate (PLP)-dependent enzyme [15]. The catalyzed reaction leads to desulfuration of L-cysteine to alanine and sulfane sulfur via the formation of an enzyme-bound persulfide (-SSH) intermediate [61]. Cysteine desulfurases can provide sulfur not only for the Fe–S cluster formation but also for other metabolic pathways (thiamine biosynthesis, for example) [15]. Eukaryotic cysteine desulfurase NFS1 operates in the so-called SDA complex [53]. SDA consists of specific desulfurase, partner protein ISD11 (LYR protein family) [45], and acyl carrier protein (ACP1) [51]. ACP1, together with the above-mentioned core Fe–S cluster-forming components, take part in generation of transient persulfides [62]. Proteins from the LYR motif family are responsible for engaging the transferring complex for the maturation of proteins with the Fe–S clusters buried, for example, succinate dehydrogenase subunit B and Rieske protein [29]. Such a connection is empowered by the co-chaperone HSC20, which can bind the transiently formed LYR–scaffold complex [63]. Furthermore, proteins of the LYR family are often associated with the Fe–S cluster synthesis for proteins of the mitochondrial respiratory complex [64]. The process of combining iron and sulfur takes place on the scaffold protein dimer ISCU2 [45] (Figure 3).

The influx of iron ions to mitochondria is supported by the carrier protein mitoferrin 1 and 2 (MFRN1/2) [45] (Figure 3). Frataxin is an iron-binding protein that acts as an iron storage; it has also been postulated that this peptide can participate in vitro as an iron donor in the Fe–S cluster synthesis [65] (Figure 3). Moreover, recent data suggest that frataxin can change the conformation of the assembly complex based on an allosteric switch and, therefore, increase the cluster formation rate [55]. The data do not rule out both functions [66]. In electron delivery, ferredoxin needs to be paired with its reductase (mitochondrial ferredoxin reductase FDXR) and nicotinamide adenine dinucleotide phosphate (NADPH) to fulfil its task [37] (Figure 3).

**Figure 4:**
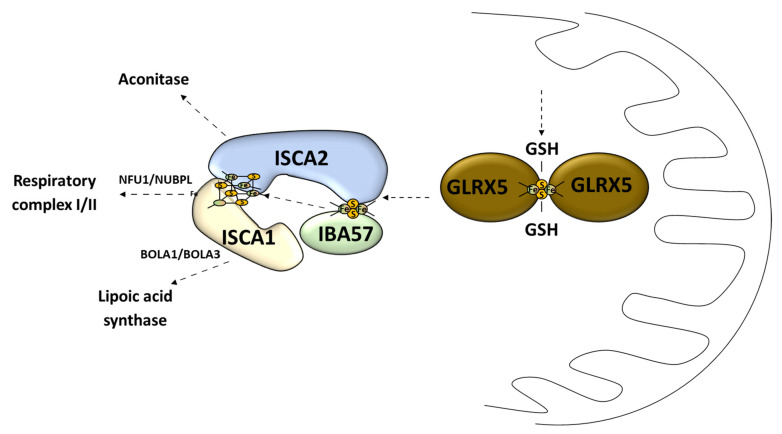
Late stage of mitochondrial Fe–S cluster synthesis. GLRX5, monothiol glutaredoxin 5; GSH, reduced glutathione; IBA57, iron–sulfur cluster assembly factor IBA57; ISCA1, iron–sulfur cluster assembly 1; ISCA2, iron–sulfur cluster assembly 2; NFU1, iron–sulfur cluster scaffold NFU1; NUBPL, nucleotide binding protein like; BOLA1, bolA family member 1; BOLA3, bolA family member 3.

This process called “early acting” leads to the formation of a [2Fe–2S] cluster intermediate that can be incorporated into the mitochondrial Fe–S protein (in cooperation with the chaperone/co-chaperone complex), sent outside of mitochondria (X–S compound), or undergo further modification (late step of mitochondrial Fe–S cluster biogenesis) [51]. Subsequently, the cluster transfer is organized by cooperating chaperons [37]. The chaperone complex composed of the HSPA9 chaperone and HSC20 co-chaperone allows for transferring the Fe–S cluster to the key transporter GLRX5 [45] (Figure 3). The homodimer complex of this glutaredoxin is known to be able to receive the Fe–S cluster directly from the cluster machinery complex by interacting with the HSPA9 chaperone [51]. It also requires two reduced glutathione (GSH) molecules [41] (Figure 4). What is interesting is that ISCU2 can donate a [2Fe–2S] cluster directly to the protein without the need of HSPA9/HSC20 [67] (Figure 4). The export of newly assembled clusters requires three different compounds: one membrane channel protein ABCb7 (yeast protein atm1, ATP-binding cassette (ABC) transporter family) [45], one sulfhydryl oxidase (FAD-linked sulfhydryl oxidase (ALR) [68], and one reducing factor (glutathione) [20].

#### 3.1.2. Late Step of Mitochondrial Fe–S Cluster Synthesis

The second stage of the Fe–S cluster formation takes place in the mitochondria where the [4Fe–4S] cluster is made of two [2Fe–2S] clusters [69]. This process is called the late-acting machinery [51]. It depends on the [2Fe–2S] cluster pass to form a working complex carried out by GLRX5 [51] (Figure 4). The late-step assembly machinery has been proposed to form the ISCA1–ISCA2–IBA57 complex that does not interfere with early stage components [51]. Recently it has been shown that this complex can convert the [2Fe–2S] cluster into the [4Fe–4S] cluster, which was demonstrated in vitro [70]. Sheftel et al. showed that the presence of all three proteins was crucial for optimal [4Fe–4S] cluster assembly and was conserved in its nature [71] (Figure 4). The association scheme of components of this pathway can be different under specific conditions, but it still remains unclear [72,73] (Figure 4). The late-acting machinery is essential in the production of the [4Fe–4S] clusters for aconitase-type protein [74], succinate dehydrogenase, radical SAM enzymes, and lipoic acid synthase [71]. The synthesis and delivery of the Fe–S cluster for specific proteins requires additional transporters: NFU1 and NUBPL in cluster formation for the respiratory complex 1; BOLA1 and BOLA3 in cluster delivery for lipoic acid synthase [75] (Figure 4).

## 4. Cytosolic Iron–Sulfur Cluster Synthesis

### 4.1. Cytosolic Fe–S Cluster Assembly Machinery

**Figure 5:**
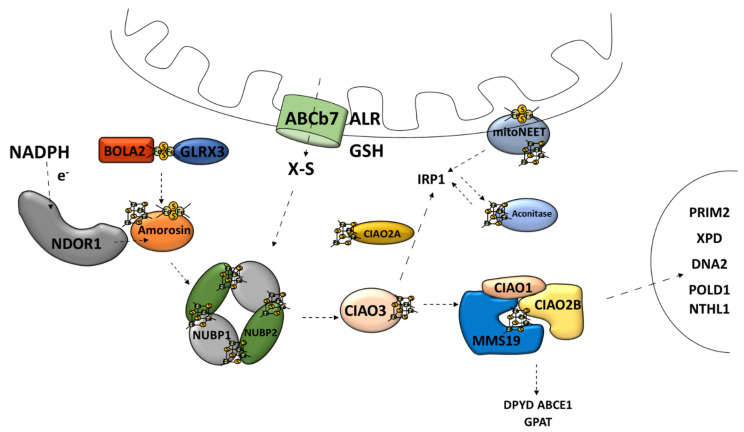
Cytosolic [4Fe–4S] iron–sulfur cluster assembly and targeting machinery. ABCb7, ATP-binding cassette (ABC) transporter family; ALR, mitochondrial FAD-linked sulfhydryl oxidase; GSH, reduced glutathione; X–S, unidentified X–S compound; NDOR1, diflavin oxidoreductase 1; Amorosin, Fe–S cluster assembly protein DRE2 homolog; BOLA2, bolA family member 2; GLRX3, glutaredoxin 3; NUBP1, cytosolic Fe–S cluster assembly factor NUBP1; NUBP2, cytosolic Fe–S cluster assembly factor NUBP2; IOP1/CIAO3, cytosolic iron–sulfur assembly component 3; CIAO1, cytosolic iron–sulfur protein assembly protein CIAO1; CIAO2B, cytosolic iron–sulfur assembly component 2B; MMS19, cytosolic iron–sulfur assembly component MMS19; Aconitase/IRP1, aconitase 1; DPYD, dihydropyrimidine dehydrogenase; ABCE1, ATP binding cassette subfamily E member 1; GPAT, glutamine phosphoribosyl-pyrophosphate amidotransferase; POLD1, DNA polymerase δ; NTHL1, DNA base-excision repair enzyme; XPD, general transcription and DNA repair factor IIH helicase subunit XPD; RTEL1, regulator of telomere elongation helicase 1; DNA2, DNA replication helicase/nuclease 2; PRIM2, DNA primase subunit 2.

The second major route of the Fe–S cluster assembly pathways is the cytosolic Fe–S cluster assembly machinery, also referred to as the cytosolic iron–sulfur cluster assembly (CIA) [30]. CIA is responsible for the Fe–S cluster assembly for cytosolic and nucleic Fe–S proteins [9]. This is a multistage process consisting of at least two stages: the nascent cluster assembly upon the scaffold complex and delivery to apo-protein [76]. The scaffold complex consists of NUBP1–NUBP2 protein [45,77] (Figure 5). Such a complex has the ability to bind two [4Fe–4S] clusters on one NUBP1 monomer [78]. The assembly process requires electrons, which are provided by diflavin oxidoreductase (NDOR1) in cooperation with amorosin from NADPH [77,79] (Figure 5). A recent observation describes an interaction between the mitochondrial and cytosolic Fe–S protein assembly machinery linked by NEET proteins (mitoNEET) in which the Fe–S cluster is coordinated by three Cys residues and one His residue [80]. It is anchored into the outer membrane of the mitochondrion with one of its parts located in the cytosol [81]. Under oxidative conditions, various connections between the mitoNEET cluster allow for the transfer of this cluster via the protein–protein interaction into the apo-form of proteins, such as the bacterial FDX [82], IRP1 [83], or amorosin [84]. Protein BOLA2 forms a protective complex with glutaredoxin 3 (GLRX3) allowing for the transfer of the newly formed [2Fe–2S] cluster from the mitochondria through the cytosol and taking part in the GLRX3–BOLA2-dependent amorosin maturation pathway [41,85] (Figure 5).

### 4.2. Cytosolic Fe–S Cluster Delivery Machinery

Transport and insertion into apo-protein of the fully assembled cytosolic Fe–S cluster are supported by the Fe–S protein named CIAO3 (human cytosolic iron–sulfur assembly component 3, also called IOP1) [86,87], in cooperation with the CIA-targeting complex (CTC) [77]. CTC is composed of the “transducing-like” protein CIAO1 [88], CIAO2B/FAM96B [77], and protein MMS19 [89]. The proteins involved in the CTC complex affect its ability to attach the Fe–S protein and provide stability to this complex [90]. The CTC complex is responsible for the maturation of many cytosolic proteins, such as dihydropyrimidine dehydrogenase (DPYD) and glutamine phosphoribosyl-pyrophosphate amidotransferase (GPAT) [77]. Furthermore, the CIA-targeting complex also transfers the Fe–S clusters to the nuclear Fe–S proteins, such as the catalytic subunit of DNA polymerase δ (POLD1), DNA base-excision repair enzyme (NTHL1), DNA helicase XPD, helicase-nuclease DNA2, and regulator of telomere length 1 (RTEL1) [19,77,91,92] (Figure 5). Moreover, CTC is responsible for the Fe–S cluster delivery for aconitase maturation [77,93].

Overall, the CIA system is very similar to ISC when it comes to the scaffold assembly process, chaperone-mediated release, and delivery pathway [37]. However, they are not evolutionarily related [77]. CIA relies on unknown sulfur-containing compounds, which have a mitochondrial origin [32]. The molecule that leaves the previously mentioned ABCb7 channel is an unidentified X–S compound [19] (Figure 5). It has been shown that mitochondria are capable of producing a specific form of intermediate Fe–S cluster (Fe–S_int_) for the CIA assembly machinery [94].

## 5. Repair of Damaged Fe–S Clusters

Enzymes that rely on the Fe–S clusters activity are endangered not only by reactive oxygen species (ROS) [10] or nitric oxide [95] but also by heavy metals [96,97] and iron shortage [15]. Unfavorable conditions can also alter sulfur bioavailability and trafficking [49]. Both the first listed factors can react with each other creating even more toxic compounds such as peroxynitrite (ONOO^−^) [98]. This molecule can easily nitrate a tyrosine residue or oxidize the thiol residue of cysteine, alternately allowing them to be changed by S-glutathionylation [99]. This activity occurs rapidly in the enzyme active site, thereby changing its conformation [100]. Nitric oxide alone rapidly reacts with cluster-forming dinitrosyl iron complexes (DNICs) and even more complicated structures [101] or is responsible for thiol nitrosylation [102,103]. The radicals NO and ^●^OH can bind to Fe atoms of the Fe–S clusters [104]. ROS, mainly superoxide (O_2_^−^), can oxidize Fe–S clusters starting with an iron release, promoting cluster instability and slow degradation leading to irreversible damage to the protein backbone of a particular enzyme [105]. Therefore, enzymes that undergo prolonged exposure to oxidative stress show a poor prognosis for renewal [106]. The formation of hydroxyl radical via Fenton reaction significantly accelerates destruction [81]. Bruska et al. performed detailed studies of a different kind of ROS–Fe–S cluster interaction [104]. Most Fe–S proteins hide the vulnerable cluster inside the polypeptide chain; however, some of them require direct involvement of the cluster to perform their activity [105]. Such exposed clusters, mainly [4Fe–4S] forms, are more prone to oxidative stress [107]. Some enzymes containing [4Fe–4S] clusters, under oxidative stress, can keep [2Fe–2S] clusters and work with lower activity [108]. Oxidative stress can devastate the function of many enzymes [106]; however, some of them may return to correct function after such an event, and some of them are highly resistant to oxidation or undergo reversible modification [101]. It depends on the composition and solidity of the Fe–S cluster [104].

### Fe–S Cluster Reconstitution Attempt

Stress oxidation agents often damage cysteine residue from the active site of important enzymes, which can be reduced in the process of repair [106]. It has been shown that inactive Fe–S clusters can be restored following the chemical or semi-enzymatic reconstitution protocol [64]. The cluster of bacterial NADH-cytochrome c reductase can be reconstituted in this way [109]. Superoxide dismutases (SODs) are enzymes actively participating in the frontline against ROS [110]. The SOD knockout *E. coli* dehydratases [4Fe–4S] cluster is rapidly damaged by ROS and undergoes reconstitution after the exposure is stopped [111]. Furthermore, the function of the aconitase [4Fe–4S] cluster, after being altered by nitric oxide, can be restored by iron, sulfide, and 1,4-dithiothreitol (DTT) after the NO influx is stopped [112]. Using the same compounds, restoration of peptide deformylase (PDF) and isopropylmalate isomerase (IPMI) of *Bacteroides thetaiotaomicron* can be performed [105,106]. The fumarate and nitrate reduction regulatory protein activity crucially depends on the process of switching forms of the cluster between [4Fe–4S] to [2Fe–2S] while sensing O_2_ and the reaction with IscS, L-cysteine, ferrous ions, and DTT can reverse it [113]. The activity of yeast proteins of the amino acids synthesis pathway: homoaconitase (Lys4p) and isopropylmalate dehydratase (Leu1p), which have the [4Fe–4S] cluster, can be restored under anaerobic conditions after superoxide-mediated inactivation [114].

The overall activity of cysteine desulfurase and its role in sulfur mobilization has been summarized [115]. Cysteine desulfurase may be involved in FNR apo-protein reparation after NO exposure [116,117]. The [4Fe–4S] cluster of endonuclease III can be reconstituted by using cysteine desulfurase (IscS), L-cysteine, ferrous ions, and DTT after exposure to NO by a new cluster assembly process in vitro [118]. On the other hand, some investigated bacteria without IscS protein were still able to repair clusters but at a slower rate (6-phosphogluconate dehydratase, fumarase A) [111]. Furthermore, cluster repair in the *IscS* mutant of *E. coli* with iron source/reducing agent (DTT) was unsuccessful in vitro [119]. Interestingly, the reconstitution of these clusters takes place without new protein synthesis de novo [111,112,114,118].

Other factors can play a crucial role in the recovery of the [Fe–S] clusters [120]. MitoNEET primarily affects the repair of the apo-form of aconitase (IRP1) and then its maturation [45]; it is also capable of donating this cluster to *E. coli* apo-ferredoxin [120]. Furthermore, di-iron proteins taking part in the Fe–S cluster synthesis are capable of fumarase A [4Fe–4S] cluster repair from *E. coli* [121], and two enzymes from yeast (aconitase B and fumarase A) [122]. Spinach apo-ferredoxin can be converted into a functional enzyme by using the *E. coli* RIC protein and IscS, DTT, and L-cysteine [123]. Furthermore, the activity of damaged clusters in cells without functioning RIC protein can be restored after RIC supplementation [124]. Interestingly, RIC proteins can be crucial factors that allow bacteria to survive in deep tissues after infection [98].

N-acetyl-L-cysteine and GSH were able to repair clusters to a much lower degree [125]. However, GSH failed to protect the *E. coli* [4Fe–4S] cluster of dihydroxyacid dehydratase from NO-mediated transformation [102].

## 6. Sulfurtransferases

**Figure 6:**
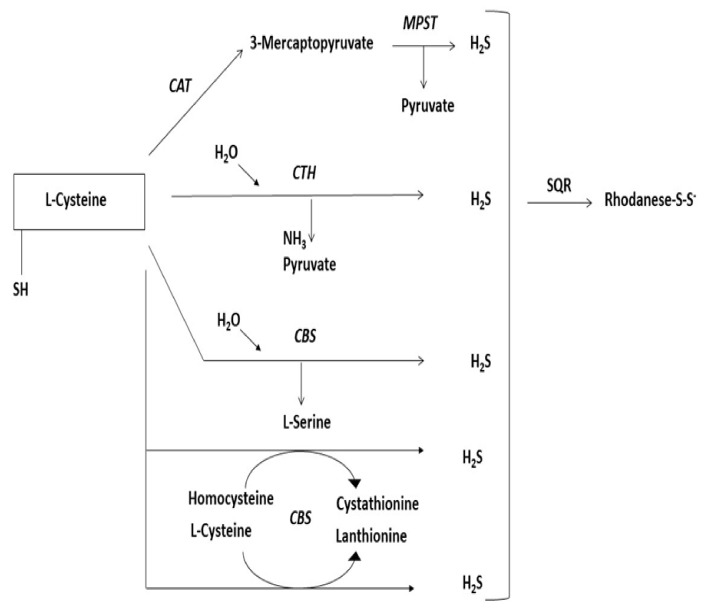
Schematic diagram showing the L-cysteine desulfuration pathway resulting H_2_S release. CAT, cysteine aminotransferase, MPST, 3-mercaptopyruvate sulfurtransferase, CTH, cystathionine γ-lyase, CBS, cystathionine-β-synthase, SQR, sulfide quinone oxidoreductase.

Sulfurtransferases are a widespread group of enzymes that can be found in archaea, bacteria, and eukaryotes [126]. They can catalyze sulfane sulfur atom transfer from a donor to a proper nucleophilic sulfur acceptor [127]. During such reactions, a persulfide-containing intermediate (enzyme-SSH) is created [128]. It is composed of at least a single catalytic rhodanese-like domain (RLD) [129], possibly two or even more [130], with specific conserved C-terminal-characteristic cysteine residue of the functional catalytic domain [131]. Furthermore, the structural localization of these domains is their specific feature [132]. Enzymes that possess such a catalytic cysteine (redox-active) play a critical role in many biological processes [133,134]. The group of enzymes involved in L-cysteine metabolism [135] are sulfurtransferases participating in the desulfuration pathway of L-cysteine, mainly 3-mercaptopyruvate sulfurtransferase (MPST, EC 2.8.1.2), thiosulfate sulfurtransferase (rhodanese or TST, EC 2.8.1.1), and lyases participating in the transsulfuration pathway of L-cysteine, mainly cystathionine γ-lyase (CTH, EC 4.4.1.1) and cystathionine-β-synthase (CBS, EC 4.2.1.22) [136] (Figure 6). CTH and CBS are PLP-dependent enzymes that belong to the group of lyases [135]. The predominant role of these enzymes is their key function in the transsulfuration pathway from L-methionine to L-cysteine [135]. On the other hand, these two enzymes take part in the further transformation of L-cysteine, whereby H_2_S production occurs [136]. Sulfurtransferases act in a two-step reaction in which sulfur is transferred to cysteine residue on the reactive site of the enzyme, where persulfide is created firstly from a suitable sulfur donor, and then sulfur is transferred to the nucleophilic acceptor [137,138]. During this process, particular complexes are formed: 1. Enzyme-SH-substrate at first, then 2. Enzyme-S-SH + product, so that ultimately 3. Enzyme-SH + S-product is formed [139]. This process is called double displacement [140]. Cysteine desulfurase (EC 2.8.1.7) shares the same mechanism of sulfur transfer [115]. According to their abilities MPST, CBS, and CTH affect the increase of and TST affects the decrease of the amount of sulfur in the sulfane sulfur pool [135]. Thus, participating in overall sulfur fraction equilibrium [141]. Malfunctions of these enzymes can result in various diseases [137,142,143,144]. Better understanding of multiple aspects of sulfurtransferases activity would give us the ability to control overall H_2_S production and its concentration-dependent action [145]. Sulfurtransferases have become promising targets for the development of new therapies [146]. Nowadays, advanced research aims at developing selective inhibitors for particular enzymes and reliable measurement tools of their overall activity [147].

### 6.1. Rhodanese and 3-Mercaptopyruvate Sulfurtransferase

Thiosulfate sulfurtransferase (rhodanese, TST, EC 2.8.1.1) and 3-mercaptopyruvate sulfurtransferase (MPST, EC 2.8.1.2) are important enzymes of the rhodanese/Cdc25 phosphatase superfamily [141]. These enzymes show high sequence similarity to each other [148]. TST is found in the mitochondria of eukaryotic cells [149]. This enzyme consists of a single polypeptide, about 32 kDa in molecular weight [139]. MPST shows both cytosolic and mitochondrial localization [150] with different isoforms (mitochondrial 35 kDa and mitochondrial/cytosol 33 kDa) existing [151]. Both proteins show specific occurrence in tissues [152,153]. Changes in this pattern are often linked with metabolic disease development and aging [138]. TST and MPST are composed of tandem rhodanese domains [128]. Cysteine localized in the enzyme active site (MPST- Cys^248^, TST- Cys^247^) is crucial for prime enzyme activity [154,155]. The sequence of amino acids located in the active site loop determines substrate specificity [155].

The best characterized bovine TST has maintained its double-domain structure and is considered to be TST sensu stricto [128]. The ability of TST that has been first discovered is to catalyze the reaction between thiosulfate and cyanide in which thiocyanate and sulfite are produced [156]. Interestingly, TST is involved in sulfane sulfur transformation into thiosulfate [157]. In this case, the initial source of sulfur is SQR acting in the process called mitochondrial sulfide oxidation, resulting in persulfide formation and electron donation on the respiratory chain [157]. This enzyme allows for incorporating H_2_S into the bound labile pool of sulfur inside the cell, thus participating in H_2_S clearance [158,159]. TST is capable of changing its conformation after oxidation, which grants this enzyme new features [160]. Moreover, phosphorylation of TST introduces a conformational change ability [161]. MPST can catalyze the reaction between cysteine-derived 3-mercaptopyruvate (3MP) and sulfur-acceptor substrate to yield pyruvate and enzyme-bound persulfide [162,163]. The enzyme is able to transfer this outer sulfur to an abundant number of small molecules or proteins [138]. Transferring sulfane sulfur onto its most suitable acceptor- thioredoxin (which ends up in the oxidized form), results in the enzyme’s active site restoration and H_2_S release [155]. 3-Mercaptopyruvate is produced from cysteine by cysteine aminotransferase (CAT; EC 2.6.1.3). Cooperation of MPST: CAT enzymatic system allows for transferring the sulfur from cysteine to active site cysteine and further to acceptor protein [164]. The transfer of sulfane sulfur is one of the possible mechanisms of persulfide species (Cys-SSH, GSSH, protein-SSH) formation [146]. Furthermore, MPST itself can generate H_2_S_n_ species (including H_2_S) [134]. In this case, the newly formed H_2_S_n_ can directly react with L-cysteine, GSH, or various proteins to create persulfide species [146]. Overall, Nagahara and colleagues summarized various reactions that can be catalyzed by MPST [154]. When MPST is unable to sustain its activity, TST expression is increased [165]. Proteins of the TST family are widespread in nature [166]. They are a heterogeneous group, differing from each other at various levels [130]. They form a diverse group of proteins called rhodanese-like proteins with identified examples divided into groups [128]. *E. coli* protein ThiI, which is a sulfurtransferase, has a similar sequence as TST [166]. The similarity also involves the amino acid sequence of the characteristic and catalytically critical “P loop” motif [167]. It seems to be only a temporary sulfur carrier between cysteine desulfurase IscS in the tRNA thiolation process [168]. *Azotobacter vinelandii* rhodanese-like protein (RhdA) can interact with *E. coli* cysteine desulfurase IscS with the use of L-cysteine, and another protein, RhdA-SSH, can carry sulfur to [2Fe–2S] holo adrenodoxin protein synthesis [129,169]. The carried sulfur is released after the apo-protein–RhdA complex formation and can be incorporated into the Fe–S cluster with iron ions in the presence of 2-mercaptoethanol [169]. Moreover, RhdA seems to modulate specific cysteine desulfurase (NifS or IscS) persulfide production, therefore, it cooperates with the enzyme during growth, increased cysteine concentration, and generally in sulfur administration [170]. However, deletion of the RhlA rhodanese-like protein from *Streptomyces clavuligerus* did not change the overall TST activity [171].

### 6.2. Participation of Rhodanese and 3-Mercaptopyruvate Sulfurtransferase in Fe–S Cluster Formation and Reconstitution

Sulfurtransferases have been proposed to be involved in the biogenesis of iron–sulfur clusters [172]. It has been shown that incorporating sulfur species into protein requires specific proteins and complex pathways [131]. Solvent-exposed clusters, which often serve as a Lewis-acid, are much more vulnerable to reactive oxygen species as H_2_O_2_, and in vivo analysis showed that only clusters undergo destruction, without damaging the specific peptide [111,173]. After a promising start, several unsuccessful attempts at recreating damaged clusters were made (using inorganic sulfide, iron, and 2-mercaptoethanol) [174]. Subsequent reports indicated that sulfurtransferases may be involved in the process of restoring the functional activity of apo-proteins [175]. TST was observed to restore the activity of succinate dehydrogenase (complex II) [175], NADH dehydrogenase [176], xanthine oxidase [177], ferredoxin of spinach [178], NADH: nitrate reductase [179], bacterial ferredoxin [180], and bacterial nitrogenase [181] (Table 1). Interestingly, the reaction rate when TST was employed was twice as fast as in the case of purely chemical synthesis [172]. More than 30% of all TST in bovine liver mitochondria is connected to its membrane where they form complexes with other compounds and may play a crucial role in the Fe–S cluster reconstitution [182], or somehow support it [175]. Moreover, MPST in cooperation with other components can also restore the activity of adrenal ferredoxin with an efficiency similar to that achieved in a chemical reaction [183]. The mechanism of such reconstitution depends on the phosphorylation/dephosphorylation continuum of TST where the dephosphorylated enzyme can insert sulfur into the newly forming Fe–S cluster [137]. Such a role is getting attention recently [184,185]. Sulfurtransferases participate in both the bound sulfane sulfur pool and acid-labile sulfur formation as well as H_2_S generating from these pools [186]. Increased H_2_S production is associated with the induction of antioxidative mechanisms [187]. H_2_S can be utilized by TST or spontaneously reacts with oxidized thiol residues, creating persulfide suitable for transfer [186]. More research is required to confirm the potential participation of the Fe–S cluster reconstitution in direct/indirect support.

### 6.3. Antioxidant Properties of Sulfurtransferases Involved in Maintaining Fe–S Cluster Function

Evidence shows that cysteine desulfurase is a primary source of sulfane sulfur for biomolecules, including the Fe–S cluster formation [131]. Mitochondria are a major cellular compartment when reactive oxygen species are produced [188]. Enzymes that possess catalytic cysteine residue of the active site are exposed to a constant attack of hydrogen peroxide [189].

#### 6.3.1. Involvement of Sulfurtransferases in Antioxidant Response

TST and rhodanese-like protein may play an antioxidant role in invertebrates [190]. Rhodanese-like protein or rhodanese homologue (*MnRDH2*) play an important role in maintaining the redox balance in invertebrates [148,191,192]. Both mammalian TST and MPST are involved in maintaining antioxidant defense [134,193]. Glutathione in the reduced (GSH) and oxidized (GSSG) forms plays a crucial role in maintaining redox homeostasis mainly by preserving sulfhydryl (-SH) group oxidation [188]. GSH in cooperation with glutathione peroxidase contributes to this process via ROS-scavenging (mainly H_2_O_2_ and lipid peroxides) [137,155]. TST takes part in the sulfide oxidation pathway inside mitochondria where sulfur is transferred from H_2_S to specific acceptors: sulfide quinone oxidoreductase (SQR) [1,162,194]. In cooperation with GSH, TST is also able to react with selenite (SeO_3_^−2^) to produce a stable intermediate (E-Se TST), and it takes part in selenium administration [195]. In addition, bovine liver TST [166] and MPST from *Leishmania* [149] have a higher affinity for the reduced form of thioredoxin than for its natural substrate, cyanide [166,170,196]. The presence of reduced thioredoxin (thioredoxin and Trx reductase system) is crucial to maintain the sulfur administration activity of TST [196] and MPST [197]. Nagahara has proposed a scheme of transformations of closely related elements (Grx, GSH, MPST) [197] and demonstrated in which way redox changes affect MPST activity step by step [154].

Interestingly, thioredoxin seems to be an important substrate for all TST proteins [198]. Moreover, thioredoxin can regulate the TST function by reducing propenyl sulfur protein (stereoisomer of S-allylcysteine, SAC) to restore the TST activity [199]. The thus formed glutathione persulfide can be used to reduce thioredoxin [137].

#### 6.3.2. External Molecules with Ability to Modulate Sulfurtransferases Activity

Overall, external stress enhances H_2_S-related enzyme activity, such as TST [200]. However, Kruger et al. observed a reduced expression and translation of TST with a high production of superoxide in the mitochondria of monocytes, which could predict mortality [201]. Radiation causes an overall oxidative stress, and in the liver, it induces an antioxidative response [193]. Such a response is also generated along with rhodanese-domain-bearing proteins in ethanol-treated murine livers and other bacterial cells [170]. Depletion of any of ISC or CIA-conserved factors results in a lower survival rate of the human embryonic kidney 293 cells (HEK 293 cell line) in response to UV radiation or methyl-methane sulfonate (MMS) exposure [89]. It is noteworthy that, in mouse liver, a low-dose radiation exposure enhances the transcription of thioredoxin mRNA and a low dose rate, while long-term radiation exposure induces TST expression for a long period of time [202]. Besides radiation exposure, there are numerous factors that enhance TST: gene expression (resveratrol [203], enzyme activity-α-lipoic acid [204]) or protein expression (*Phellinus linteus* polysaccharide extracts) [205]. The activity of TST and MPST is increased after garlic-derived diallyl trisulfide (DATS) treatment [206]; moreover, N-acetyl-L-cysteine increases the MPST activity [207]. On the other hand, the level of TST and MPST proteins in the mitochondria is decreased after 4-hydroxybenzyl isothiocyanate (HBITC) (H_2_S donor) treatment [208]. The activity of all these factors is associated with the TST antioxidative activity [202]. Interestingly, another chemical compound of garlic, i.e., sodium 2-propenyl thiosulfate, can interact with the active site of TST, inhibiting its activity and expression [199].

Pagani et al. showed that lipoic acid and its reduced form (dihydrolipoic acid) affected TST activity [209] with a relatively low affinity [210]. The reduced form decreased the activity of TST after preincubation, but on the other hand, the oxidized form had no effect on the enzyme activity [209,210]. This is explained by the fact that dihydrolipoate is a sulfur acceptor from TST [210]. Moreover, the presence of thiosulfate inhibits decreasing TST activity [209]. Proteins that possess fully functional Fe–S clusters (holoproteins) may decrease rhodanese activity [181].

Oxidative stress is linked with many cancers [211]. Downregulation of TST expression is associated with many tumors [212,213,214]. Deficiency in TST activity is often reported in the case of malignant cells [215]. Some cancer cells can promote the expression of H_2_S-producing enzymes, such as MPST, to control oxidative stress [216]. Overexpressed protein Naf-1 with the labile Fe–S cluster is used by tumor cells to increase their aggressiveness by enhancing proliferation and tolerance to oxidative stress. This activity can be stopped, and it leads to ROS, iron, and oxidative stress accumulation [211]. The proliferation- and expansion-resistant cancer type can be stopped by induction and sensitization to oxidative stress [217]. In response to the generation of reactive oxygen/nitrogen/sulfur species (ROS/RNS/RSS), the protein cysteine thiols (R–SH) undergo a range of oxidative modifications, for example: nitrosylation (R–SNO), sulfenylation (R–SOH), and persulfidation (S-sulfuration, R–SSH) [218,219]. The enzymes that possess catalytic cysteine residues of the active site are exposed to a constant attack by hydrogen peroxide [189]. Nagahara showed that reactive oxygen species could oxidize the catalytic site Cys^247^ of MPST (Cys–SO^−^, Cys–SO^2−^ and Cys–SO^3−^) and that the MPST activity decreased under oxidizing conditions and increased under reducing conditions [220]. Our previous studies [208] have demonstrated that the increased H_2_S and thiosulfate levels in HBITC-treated SH-SY5Y cells have been associated with downregulation of the level of TST and MPST, which suggest that the sulfhydryl groups of these enzymes can be modified by S-sulfuration (-SH to -SSH), or by oxidative stress (-SH to -SOH).

## 7. Conclusions

Sulfur is a very important microelement. We can distinguish different pools with specific abilities and functions. Acid-labile sulfur is a part of the iron–sulfur clusters, which are included in different proteins where they can perform various tasks. Mitochondria are the primary place where the Fe–S clusters are assembled. The source of sulfur is the cysteine desulfurase reaction. The mitochondrial process can be divided into two steps: the [2Fe–2S] cluster synthesis and [4Fe–4S] cluster synthesis. Interestingly, the mitochondrial machinery can produce an unidentified sulfur compound, X–S, which after being transferred to the cytoplasm may be a source of sulfur for the cytosolic Fe–S cluster formation machinery. Biological sulfur transfer via the persulfide group (containing sulfane sulfur atom) seems to be crucial for the Fe–S cluster formation. Many reports suggest the involvement of sulfurtransferases in the Fe–S cluster formation or repair/reconstitution, as well as sulfur-containing enzymes’ modification [221]. The exact mechanism is poorly understood. According to Freibert and colleagues, proven procedures were developed to reconstitute the Fe–S clusters by chemical reconstitution or de novo synthesis [64]. Many papers show that bacterial cysteine desulfurase (IscS) can repair damaged clusters; however, the repair occurs at a slower rate as compared to that in wild-type cells [111]. Agents of oxidative stress are often able to damage cysteine residues of the active site of enzymes. Reduction of oxidized cysteines can be performed by DTT or other agents, such as thioredoxin or glutaredoxin. The very first research addressing this topic has shown that it is unlikely that TST and MPST are responsible for the formation of the Fe–S clusters, but they cannot be excluded [183]. Fe–S clusters are oxidative-stress-sensitive structures. During cell life, oxidative stress is common, therefore the Fe–S clusters are often damaged. During the catalyzed reaction, both TST and cysteine desulfurase create cysteine persulfide, primarily in active sites, then they donate sulfane sulfur atoms to an acceptor molecule. Sulfurtransferases (MPST and TST) are involved in the antioxidative mechanism of cells. TST plays a protective role in oxidative stress induced by many factors, including radiation. Oxidative stress damages the Fe–S cluster and is often linked to cancer formation; therefore, it can be a potential target of treatment. MPST and TST, but mostly TST, seem to non-directly participate in Fe–S cluster formation; however, reports show possible direct participation in reconstitution/repair. The involvement of MPST and TST in oxidative stress could also provide an indirect mechanism of Fe–S cluster protection. Nevertheless, the synthesis, maturation of the Fe–S protein, repair of the Fe–S cluster, and antioxidative mechanism to protect clusters are not fully clarified.

## Figures and Tables

**Table 1:** List of enzymes that undergo a process of reconstitution of their activity with the participation of TST and MPST.

Protein Name	Key Components Used for Reconstitution	Measurement	Outcome of Reconstitution	Reference
Succinate dehydrogenase (EC 1.3.99.1)	TST and thiosulfate	Measurement of labeled sulfane sulfur	Increased radioactivity of succinate dehydrogenase	Bonomi et al., 1977
Spinach ferredoxin [2Fe–2S] cluster	TST, thiosulfate and iron ions (Fe^3+^)	Yield of reconstitution (%)	Reconstitution of ferrodoxin’s activity	Pagani et al., 1984
NADH dehydrogenase	TST and thiosulfate	Measurement of labeled sulfane sulfur	Increased radioactivity of NADH dehydrogenase	Pagani and Galante, 1983
Xanthine oxidase	TST, thiosulfate, and sulfhydryl reagent	Measurement of labeled sulfane sulfur	Increased radioactivity of xanthine oxidase	Nishino et al., 1983
NADH-nitrate reductase	TST and thiosulfate	Yield of reconstitution (%)	Reconstitution of NADH-nitrate reductase’s activity	Tomati et al., 1976
[4Fe–4S] cluster of bacterial ferredoxin	TST and thiosulfate	Yield of reconstitution (%)	Prominent reconstitution of ferrodoxin’s activity	Bonomi et al., 1985
Nitrogenase of *Klebsiella pneumoniae*	TST, thiosulfate and iron ions (Fe^3+^)	Change of original activity (%)	Restoring two-thirds of the original activity	Pagani et al., 1987
Adrenal ferredoxin	3-Mercaptopyruvate sulfurtransferase, 3-mercapto- pyruvate, ferrous ions	Change in absorbance at 414 nm	Increase in absorbance at 414 nm	Taniguchi and Kimura, 1974
